# Epidemiological Correlation of Pulmonary *Aspergillus* Infections with Ambient Pollutions and Influenza A (H1N1) in Southern Taiwan

**DOI:** 10.3390/jof7030227

**Published:** 2021-03-19

**Authors:** Jien-Wei Liu, Yee-Huang Ku, Chien-Ming Chao, Hsuan-Fu Ou, Chung-Han Ho, Khee-Siang Chan, Wen-Liang Yu

**Affiliations:** 1 Division of Infectious Diseases, Department of Internal Medicine, Kaohsiung Chang Gung Memorial Hospital, Kaohsiung 83301, Taiwan; 88b0@cgmh.org.tw; 2 Chang Gung University College of Medicine, Taoyuan 333323, Taiwan; 3 Department of Intensive Care Medicine, Chi Mei Medical Center, Liouying, Tainan 73657, Taiwan; althrisas@gmail.com (Y.-H.K.); ccm870958@yahoo.com.tw (C.-M.C.); 4 Department of Intensive Care Medicine, Chi Mei Medical Center, Chiali, Tainan 72263, Taiwan; Iamkiu@gmail.com; 5 Department of Medical Research, Chi Mei Medical Center, Tainan 71004, Taiwan; ho.c.hank@gmail.com; 6 Department of Hospital and Health Care Administration, Chia Nan University of Pharmacy & Science, Tainan 71710, Taiwan; 7 Department of Intensive Care Medicine, Chi Mei Medical Center, Tainan 71004, Taiwan; kheesiangchan@gmail.com; 8 Department of Medicine, School of Medicine, College of Medicine, Taipei Medical University, Taipei 11031, Taiwan

**Keywords:** aspergillosis, fungus, influenza A (H1N1), PM_2.5_, PM_10_, pollution

## Abstract

An increase in fungal spores in ambient air is reported during a spike in particulate matter (PM_2.5_ and PM_10_) aerosols generated during dust or smog events. However, little is known about the impact of ambient bioaerosols on fungal infections in humans. To identify the correlation between the incidence of pulmonary aspergillosis and PM-associated bioaerosols (PM_2.5_ and PM_10_), we retrospectively analyzed data between 2015 and 2018 (first stage) and prospectively analyzed data in 2019 (second stage). Patient data were collected from patients in three medical institutions in Tainan, a city with a population of 1.88 million, located in southern Taiwan. PM data were obtained from the Taiwan Air Quality Monitoring Network. Overall, 544 non-repeated aspergillosis patients (first stage, *n* = 340; second stage, *n* = 204) were identified and enrolled for analysis. The trend of aspergillosis significantly increased from 2015 to 2019. Influenza A (H1N1) and ambient PMs (PM_2.5_ and PM_10_) levels had significant effects on aspergillosis from 2015 to 2018. However, ambient PMs and influenza A (H1N1) in Tainan were correlated with the occurrence of aspergillosis in 2018 and 2019, respectively. Overall (2015–2019), aspergillosis was significantly correlated with influenza (*p* = 0.002), influenza A (H1N1) (*p* < 0.001), and PM_2.5_ (*p* = 0.040) in Tainan City. Using a stepwise regression model, influenza A (H1N1) (*p* < 0.0001) and Tainan PM_10_ (*p* = 0.016) could significantly predict the occurrence of aspergillosis in Tainan. PM-related bioaerosols and influenza A (H1N1) contribute to the incidence of pulmonary aspergillosis.

## 1. Introduction

Particulate matter (PM) is complex mixture of both organic and inorganic particles, including microorganisms. Of note, fungal spores (e.g., *Aspergillus* spores) have been widely reported to be part of ambient bioaerosols in PM. Conventionally categorized PMs include PM_2.5_ and PM_10_ (mean aerodynamic diameters ≤ 2.5 μm and ≤10 μm, respectively). The measurement unit of PM is generally micrograms per cubic meter (µg/m^3^). *Aspergillus fumigatus* is an airborne saprophytic fungus. The conidia released into the earth’s atmosphere have a diameter small enough (2 to 3 μm) to settle onto microenvironments in the small particles of PM_2.5_ or PM_10_. Quantitative evaluation of fungal exposure is often conducted by analysis of the composition of fungal spores in air samples and calculation of the concentrations afterward [1,2,3,4].

Ambient *Aspergillus* spore counts were found to significantly increase from 247 spores/m^3^ in air pollutant PM_10_ of 84 μg/m^3^ to 975 spores/m^3^ in PM_10_ of 103 μg/m^3^ during dust events between December 2000 and April 2001 in Tainan, a city with a population of 1.88 million located in southern Taiwan [1]. An increase in the burdens of *Aspergillus fulmigatus* in air pollutants PM_2.5_ and PM_10_ was reported during a smog event in Beijing [2]. Despite the well-documented parallel increase in fungal spores in ambient air and PMs (PM_2.5_ and PM_10_) generated in dust or smog events [1,2,3,4], little is known about the impact of ambient bioaerosols with airborne fungal spores on pulmonary aspergillosis in humans.

Unlike classical invasive pulmonary aspergillosis (IPA) with pulmonary cavitary lesions in the severely immunocompromised patients, higher chances of IPA in patients with clinically severe influenza were reported to have various types of pulmonary lesions [5,6,7,8,9]. IPA could also occur in patients with modified immune impaired disorders or in critically ill patients, who are potentially vulnerable to IPA [10]. Because of the presence of 17% of IPA in patients suffering from clinically severe influenza in Tainan [8] and a prior high-level ambient PM_2.5_ for 2 months (>54 μg/m^3^ over 100 h per month), we previously proposed that there was a correlation between ambient air pollution and increased suspended PM_2.5_ and the soaring incidences of IPA in severe influenza patients [9]. Therefore, we designed this current study to analyze the trend of monthly total IPA cases based on presumably stable numbers of monthly administered patients in our intensive care units (ICUs) [9]. We also used % of IPA in the severe (hospitalized in ICU) influenza patients, which could be comparative to published data [5,6,7,8]. The goals of this study were to document the epidemiological linkage between air pollution and IPA patients of all hosts, but not limited to the influenza patients only. We hypothesized that IPA patients would increase in the seasons of higher PM bioaerosols and during an influenza epidemic. Elucidation of this correlation may help public health authorities to map out a plan to mitigate IPA, especially in dust events and influenza seasons.

## 2. Materials and Methods

We included and analyzed patients with IPA and patients with confirmed influenza admitted between 2015 and 2019 at any of the three institutions of Chi Mei medical systems scattered in different districts in Tainan City, including Chi Mei Medical Center (1278-bed tertiary referral teaching medical center), Chi Mei Medical Center, Liouying (876-bed regional teaching hospital), and Chi Mei Medical Center, Chiali (333-bed local teaching hospital). These facilities were located at least 40–50 Km apart from each other. A two-phase analytical study was conducted, with (1) phase 1, where patients admitted between January 2015 and December 2018 were retrospectively included for analysis, and (2) phase 2, where patients admitted between January and December 2019 were prospectively included in an observational study. Laboratory data were obtained from the electronic resources of the Chi Mei medical systems.

IPA was defined as a concurrent pneumonia and positive test for *Aspergillus* galactomannan (GM) antigen from serum, bronchoalveolar lavage fluid (BAL) and/or endobronchial secretion. Pneumonia was defined as mentioned elsewhere [11]. GM antigen was detected using Platelia *Aspergillus* Ag EIA (Bio-Rad Laboratories, Marnes-La-Coquette, France), where a cut-off value ≥ 0.5 indices indicated a positive result [10]. The IPA patients were not limited to any host factor, modified from the newly updated consensus definitions of invasive fungal disease from the European Organization for Research and Treatment of Cancer (EORTC) and the Mycoses Study Group Education and Research Consortium [12], which proposed probable IPA requiring the presence of at least one host factor (immunocompromised patients only), a clinical feature (computed tomography image), and mycologic evidence. The updated EORTC consensus defined positive GM antigen for any 1 of the following: ≥1.0 for single serum or plasma, ≥1.0 for BAL fluid, and single serum or plasma ≥ 0.7 plus BAL fluid ≥ 0.8 [12]; whereas we enrolled all IPAs in immunocompromised and non-immunocompromised hosts including severe influenza patients, who had pneumonia and a GM antigen ≥ 0.5 for serum, BAL, and/or endobronchial secretion.

Diagnosis of influenza was made based on a throat swab positive for influenza test using one of the following polymerase chain reaction (PCR) analyses: for influenza A, influenza A(H1N1), influenza A(H3N2), or influenza B. Severe influenza referred to any clinically severe influenza infection making intensive monitoring and advanced supportive care indicated for the affected patient admitted to an ICU. Severe influenza is a notifiable disease in Taiwan and is required by law to be reported to Taiwan CDC, where the influenza A should be subtyped to determine whether it is H1N1 or H3N2. Non-typed influenza A was applicable to those not requiring ICU admission and there is no need for a CDC report.

Information regarding monthly mean values of PMs that indicated levels of air pollution for differing cities/districts in Taiwan was retrieved from the Taiwan Air Quality Monitoring Network, which monitors the long-term trend of air quality from 60 general monitoring stations throughout Taiwan run by the Environmental Protection Administration, Executive Yuan. General monitoring stations were installed at populous sites or sites that are prone to higher pollution or can represent the distribution of air quality in a larger area, so that the data collected can reflect the air quality status of people’s daily lives (https://airtw.epa.gov.tw/ENG/default.aspx, accessed on 16 January 2021).

The trend of variables and the slope difference in comparison were calculated using the Theil-Sen trend test (http://www.singlecaseresearch.org/calculators/theil-sen, accessed on 16 January 2021). Spearman’s test and logistic regression were used to measure the correlation and prediction between two variables, respectively. SAS 9.4 for Windows (SAS Institute, Inc., Cary, NC, USA) was used for statistical analyses.

## 3. Results

### 3.1. Trend Analysis

Overall, 544 non-repeated IPA patients (first stage, *n* = 340; second stage, *n* = 204) were identified from 2015 to 2019. These patients were all putative/probable IPA according to the modified AspICU algorithm proposed by Schroeder et al. [10]. Positive *Aspergillus* GM tests were mainly based on positivity for blood samples (*n* = 369, 68%), endobronchial secretions (*n* = 88, 16%), BAL (*n* = 81, 15%), and endobronchial fungal cultures (*n* = 6, 1%). Positive non-blood samples were not calculated if blood samples were positive. Positive *Aspergillus* cultures were not calculated if other samples were positive. Among 204 IPA patients in the observational prospective study in 2019, the proportion of positive sample was: 54.2% (103/190) for blood, 85.5% (106/124) for endobronchial secretion, and 78.3% (18/23) for BAL fluid. The proportion of GM antigen ≥1.0 in positive samples was 51.5% (53/103) for blood, 65.1% (69/106) for endobronchial secretion, and 55.6% (10/18) for BAL fluid.

Table 1 presents trends of all different time periods (2015–2016, 2015–2017, 2015–2018, and 2015–2019), and compares 2015–2017 vs. 2018–2019. There were significant increases in 2015–2018, 2018–2019, and 2015–2019. These data highlighted that the increase from 2018 to 2019 is due to the prospective study, but IPA had also increased from 2015 to 2018.

**Figure 1:**
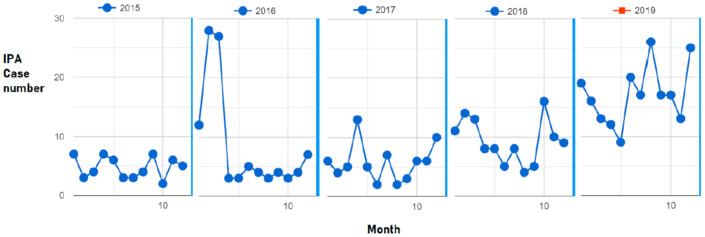
Invasive pulmonary aspergillosis (IPA)cases per month in 2019 are significantly higher than those in 2015–2018 and the trends of IPA cases show a significant increase from 2018 to 2019 (slope, 0.500, *p* = 0.005) and 2015 to 2019 (slope, 0.185; *p* < 0.00001).

Except for a spike in IPA cases found in early 2016, IPA monthly cases in 2019 (mean = 17.0, SD = 5.05) were the highest during 2015–2019 (mean = 9.07, SD = 6.73, *p* < 0.0001, Table 1). The trend of IPA significantly increased from 2015 to 2019 (slope, 0.185; *p* < 0.00001), with the most significant increase from 2018 to 2019, thus leading to a significant change between the trends of 2015–2017 and 2018–2019 (slope difference, −0.5 and *p* = 0.005). The trend in IPA within each year did not change significantly (Table 1), but a dynamic variance existed and was often the highest in the winter-spring seasons and lowest in the summer seasons (Figure 1).

The secular trend (2015–2019) of monthly average of PM_2.5_ in Tainan City insignificantly decreased (*p* = 0.3387). However, the trend of monthly cases of influenza A (H1N1) significantly increased (slope, 0.0357; *p* = 0.001) from 2015 to 2019 (see Supplementary Figure S1).

### 3.2. Correlation Analysis

#### 3.2.1. First Stage (2015–2018)

Although influenza A (H1N1) influenza circulated predominantly in 2016, a yearly-basis increase in IPA cases was not found in 2016 (*p* = 0.111), but influenza A (H1N1) had a significant correlation with the 4-year secular trend of IPA from 2015 to 2018 (*p* = 0.042). Overall influenza, non-typed influenza A, influenza A (H3N2), and influenza B had no significant correlation with the secular trend of IPA in 2015–2018 (Table 2). The air pollution in Tainan has no statistically significant correlation with % of IPA in severe influenza patients (13.0%, 41/315) during the 48-month period (Table 3).

**Figure 2:**
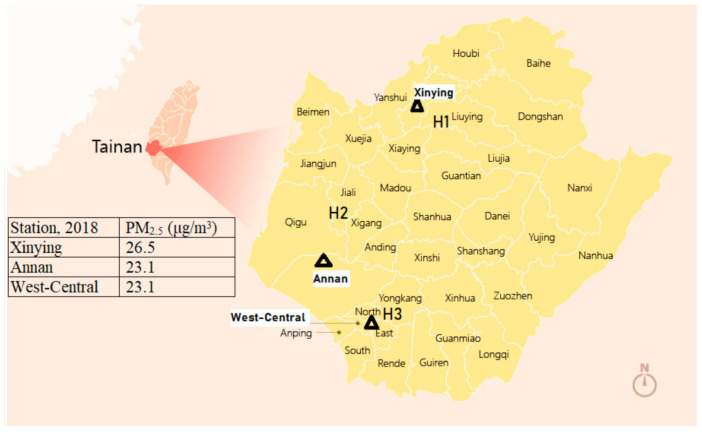
A map of Tainan City with the closest reporting station to each hospital: Xinying station closest to Chi Mei Medical Center, Liouying (H1); Annan station closest to Chi Mei Medical Center, Chali (H2); and West-Central station closest to Chi Mei Medical Center, Tainan (H3), showing no significant difference of PM_2.5_ among the three zones in Tainan.

Ambient air pollution (PM_2.5_ and PM_10_) levels in Tainan City was significantly correlated with IPA in 2018 solely, and with a longer time interval from 2015 to 2018 (Table 2). There were similar annual average concentrations of PM_2.5_ among the three zones of the closest reporting station to each hospital in Tainan City (Figure 2). The gradient of local ambient PM_2.5_ concentration was generously highest in southern Taiwan, moderate in central Taiwan, lower in northern Taiwan and lowest in eastern Taiwan (like the data in 2018, see Supplementary Figure S2A). Tainan IPA, typically in 2018, was correlated significantly with ambient PM_2.5_ of local areas (such as Tainan West-Central District and Zuoying District) in southern Taiwan, extending to the Xitun District of Taichung City in central Taiwan, but was not correlated to ambient PM_2.5_ in more distant areas, such as northern and eastern Taiwan, with an interface in central Taiwan (see Supplementary Figure S2B). Tainan IPA was also significantly correlated with PM_10_ at Tainan (West-Central area), Xinying, and Zuoying areas in 2018 (see Supplementary Figure S3).

#### 3.2.2. Second Stage (2019)

Influenza A (H1N1), but not air pollution in Tainan, was significantly correlated with the occurrence of IPA. Overall influenza, non-typed influenza A, influenza A (H3N2), and influenza B had no significant correlation with the occurrence of IPA in 2019 (Table 2). Air pollution in Tainan had no statistically significant association with % of IPA in severe influenza patients (21.7%, 20/92) during the 12-month period (Table 3). The % of IPA in severe influenza during the prospective stage was significantly higher than that of the retrospective stage (21.7% vs. 13.0%, *p* = 0.039, chi-square statistic test).

#### 3.2.3. Overall (2015–2019)

IPA was significantly linked to all influenza (*p* = 0.002), influenza A (H1N1) (*p* < 0.001), and PM_2.5_ (*p* = 0.040) in Tainan City (Table 2). The air pollution in Tainan has no statistically significant association with % of IPA in severe influenza patients (15.0%, 61/407) during the 60-month period (Table 3). The ambient PM_2.5_ in areas other than Tainan City was not correlated with Tainan IPA (see Supplementary Figure S4). The PM_2.5_ gradient in Taiwan was usually higher in the southern area than in the central area and lower in the northern area (lowest in the eastern area, not shown).

**Figure 3:**
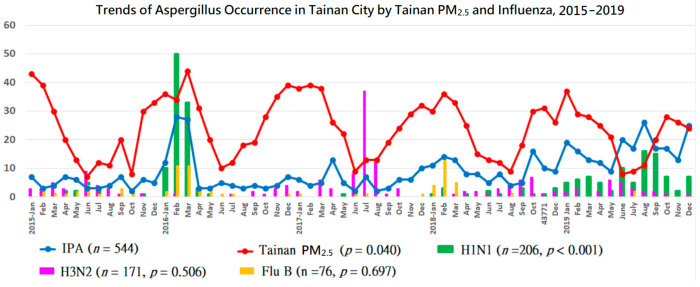
From 2015 to 2019, Tainan IPA monthly cases (blue curve) are positively correlated with local PM_2.5_ (μg/m^3^) in Tainan (red curve) and influenza A (H1N1), but have no statistically significant correlation with influenza A (H3N2) and influenza B. A surge in IPA commonly occurs in the influenza epidemic during the peak period of PM_2.5_ in spring seasons (January-March), except an autumn flu (July-September) during a low PM_2.5_ period and an unusual influenza A (H1N1) epidemic in 2019. The vertical axis represents the monthly case number for each variable of IPA, Influenza A (H1N1), influenza A (H3N2), and influenza B, as well as the monthly average concentration of Tainan PM_2.5_ (µg/m^3^). The *p* values refer to Spearman’s correlation between IPA and each variable. Tainan PM_2.5_ represents data from West-Central station.

The peak of PM_2.5_ usually occurred in the spring and the trough occurred in summer, generally compatible with the trend of case number of IPA (Figure 3). The monthly case number of IPA usually peaked in the spring, compatible with the peak seasons of PM_2.5_. An unusual IPA surge occurred in autumn 2019 during the lowest levels of PM_2.5_, while an influenza (H1N1) epidemic unusually occurred in the same season (Figure 3). The IPA trend had no statistically significant correlation with influenza A (H3N2) and influenza B. For example, a summer epidemic influenza (H3N2) occurred during the trough levels of PM_2.5_ in 2017, but it was not accompanied by an IPA surge (Figure 3).

The effects of influenza and its subtypes on the occurrence of all IPA cases showed dynamic variation each year (Table 2). IPA was not correlated with influenza (all types) and subtypes in 2015 and 2018, but was positively correlated with a large epidemic of influenza (all types) in 2016 (*p* = 0.039). The development of IPA was negatively correlated with non-typed influenza A in 2017, with a correlation coefficient (*r_s_*) of −0.596 (*p* = 0.041). Influenza A (H1N1) was positively correlated with IPA in the years 2015–2018 (*p* = 0.042), 2019 (*p* = 0.015), and 2015–2019 (*p* < 0.001). For influenza-associated IPA, % of IPA in severe influenza patients has a strong correlation with % of IPA in each subtype of severe influenza during the retrospective stage and overall period in 2015–2019 (Table 3).

### 3.3. Regression Analysis

#### Predictors for IPA (2015–2019)

After exclusion of the less relevant observed factors by univariate analysis, three models were used to establish the predictive factors of IPA (Table 4). The best model is Model A, which adopts stepwise to choose the prediction model of the relevant factor, with 68.60% of the explanatory power. Influenza A (H1N1) significantly predicted IPA (*p* < 0.0001). PM_10_ in Tainan is also a significant predictor of IPA (*p* = 0.016), but PM_2.5_ only reached a trend (*p* = 0.085). However, non-typed influenza A is a negative predictor for IPA (β = −0.44, *p* = 0.0302), which might be explained by the non-ICU influenza patients without further influenza subtyping in clinical practice. Non-severe influenza patients were less likely to develop IPA.

## 4. Discussion

Influenza-associated IPA has emerged as a major healthcare challenge globally [13]. IPA might occur in up to 16–23% of severe influenza patients [5,6,7,8], similar to our current study of 13.0% during the retrospective stage and 21.7% in the prospective stage. Epidemiological association of IPA with PM_2.5_ and severe influenza (2015–2016) has been postulated in southern Taiwan, according to prior high-level PM_2.5_ exposure before a large influenza epidemic in Tainan City [9]. Higher levels of PM_10_ in ambient air during sandstorms were accompanied by higher concentrations of *Aspergillus* spores, as reported in Tainan City [1].

Sandstorm events often occur during the northeast monsoon season in Taiwan. By long-distance transport, the northeast monsoon originating from the Asian continent could transport PM_10_ and PM_2.5_, passing through Taiwan (from north to south) to downstream southern areas from autumn to the following spring annually [14,15]. Sandstorm dust is a prolific source of PM_10_, PM_2.5_, and bioaerosols including *Aspergillus*, other fungi, and bacterial isolates [14,15,16]. Therefore, urban air pollution depends greatly on seasonality and monsoons [17]. Concomitantly with the monsoon, the concentration of these potential health-risk bioaerosols has been higher in the receptor area (such as Tainan city in southern Taiwan) than in the imported area (such as Taipei City in northern Taiwan), due to the wake-flow effect with the slowdown of wind speed, resulting in high concentrations of PM_2.5_ in southern Taiwan, moderate concentrations in central Taiwan, and low concentrations in northern and eastern Taiwan (Taiwan Air Quality Monitoring Network, https://airtw.epa.gov.tw/ENG/default.aspx accessed on 26 January 2021). However, the real impact of PM-associated bioaerosols on human fungal infections was not clearly delineated. A key aspect of limiting the fungus thread is first to ensure accurate correlation of fungal infection with PM pollution for those infected. Understanding the effects of ambient PM bioaerosols on IPA is essential for providing comprehensive medical care for these infected patients. In our current data, the IPA surge commonly occurred in spring during the northeast monsoon seasons with the highest levels of ambient PM_2.5_, implying that PM_2.5_ could be regarded as an indirect indicator of *Aspergillus* spore levels.

In this study, we first demonstrated that the trend of IPA has a statistically significant correlation with ambient PM_2.5_ and influenza (especially H1N1) in a secular 5-year data analysis. Because the incidence of IPA might be influenced by the motivation of physicians to initiate *Aspergillus* antigen testing, we conducted a prospective observational study to collect data in 2019 to compare with a retrospective study collecting data from 2015 to 2018. Overall, 544 IPA patients were identified, including 340 and 204 patients in the retrospective 4-year and prospective 1-year stages, respectively, suggesting a higher diagnostic motivation in 2019 (mean, 17 per month) than previously (*p* < 0.0001).

We used a cross-sectional sample of annual data and found a correlation in dynamic variance. In 2018, air pollution (PM_2.5_ and PM_10_) rather than influenza primarily affected the IPA trend. The ambient PM_2.5_ levels from Zuoying in Kaohsiung City through Tainan City north to Xitun of Taichung were significantly correlated with IPA in Tainan. However, the PM_10_ levels from only the relatively restricted southern areas (Tainan City, Xinying District, and Zuoying District) were significantly correlated with IPA in Tainan. These data suggest that PM_2.5_ in the broadened area and PM_10_ in the restricted local area could affect IPA in Tainan City. Thus, we might construct a risk map indicating the vulnerability of different areas to *Aspergillus* infection in Taiwan.

In contrast, in 2019, the role of influenza A (H1N1) in Tainan City surpassed the roles of PM_2.5_ and PM_10_ in the correlation with Tainan IPA. The roles of PMs were probably confounded by autumn influenza A (H1N1) accompanied by an IPA surge during a lower PM season. The trends of IPA by influenza (all types), influenza A (H1N1), and PM_2.5_ were significant in the combined two stages of the study (2015–2019).

Our data supported the dynamic effects of influenza on the incidence of IPA [18]. Schwartz et al. reported that the effect of influenza on IPA might not be universal as the incidence of influenza-associated IPA varied (0–23.1%) in different influenza seasons [18]. We expanded the dynamic effects to bioaerosols (PM_2.5_ and PM_10_), which might alternatively replace the role of influenza in the IPA incidence in some seasons or years, and vice versa. This phenomenon could be best presented by the different effects in 2018 and 2019. Air bioaerosols (PM_2.5_ and PM_10_) in Tainan City were significantly correlated with IPA in 2018, whereas influenza A (H1N1) in Tainan City reached a statistically significant correlation with Tainan IPA in 2019.

Throughout the two stages (2015–2019), IPA in Tainan was significantly linked to influenza (all types), influenza A (H1N1), and PM_2.5_ in Tainan City. Furthermore, using a stepwise regression model, influenza A (H1N1) and PM_10_ in Tainan City could significantly predict the occurrence of Tainan IPA. The results were consistent with a previous study in Tainan, which demonstrated a higher number of *Aspergillus* fungal spores in Tainan ambient air when coming across a season with a higher level of PM_10_ [1], and we further linked the PM_10_ to predict the development of IPA diseases. Furthermore, our data support a hypothesis that all the subtypes of severe influenza play a risk role for influenza-associated aspergillosis, as a strong correlation between them. However, the % of influenza-associated aspergillosis did not reach the statistical significance of its correlation with PM_2.5_ and PM_10_. Therefore, IPA of all hosts, not limited to influenza patients, increased in a season of a large influenza epidemic or influenza A (H1N1) predominance, indicating other environmental variables in the influenza seasons that may affect the incidence of IPA, such as co-circulation of other respiratory viruses or respiratory pathogens, concurrent air pollution, or increasing search for IPA by physicians. These secular data suggest that ambient air pollution (PM_2.5_ or PM_10_), as well as influenza factors (particularly H1N1 predominant seasons) can contribute alternatively or synergistically to the disease development of IPA. Research analyzing the risk hosts vulnerable to IPA in the pollution or influenza seasons is ongoing.

Our study shows its ability to detect risk factors for *Aspergillus* infections effectively, a much-needed tool for early screening of this fungus, particularly in high-risk seasons. Low motivation for testing GM for fungal infection in patients after severe influenza has been noticed outside Europe [19,20]. On the contrary, high vigilance of physicians to test the GM assay increased the detection of aspergillosis during the coronavirus disease 2019 epidemic in Taiwan [21]. In the current study, we identified the additional environmental risks of IPA. These critical events might afford aid for healthcare systems in starting up deployments (such as setting up an “IPA Watch” system and public education for medical mask wearing) to combat and prevent the fungal disease early on. Whether prevention measures are effective for fungal disease control also needs further investigation.

A limitation of this study might include the possibility of false-positive *Aspergillus* antigen testing and increasing the diagnostic motivation of physicians in the prospective observation study, and data retrieved from retrospective analysis in 2015–2018 that might underestimate the incidences of IPA. A positive GM index in serum (>0.5) or in BAL (≥1.0) has been proposed for influenza-associated pulmonary aspergillosis [22]. It might be more appropriate to use GM index ≥1.0 in BAL for initiation of anti-fungal treatment based on the cost-effective evaluation [12]. Tests for GM in BAL and serum samples at a cutoff index ≥0.5, aiming to increase test sensitivity for critically ill patients, were proposed by Schroeder et al. [10]. Besides, a higher GM index in lung excreta of ≥1.88 might be a much better predictor of IPA compared to serum GM [23]. Our simplified GM cutoff index ≥0.5 in serum, BAL, or endobronchial secretions could not support initiation of antifungal therapy, which might need an individualized or pragmatic approach. The main goal of the study was to assess the long-term trend of IPA influenced by the air pollution and influenza epidemic. The secular trend difference would not be influenced if a cut-off value has been constant. However, our data, including secular trends with different seasons, PM bioaerosols, and influenza factors, provide comprehensive epidemiology of risks for IPA to support early diagnosis of the fungus and continuous prospective monitoring of these environmental impacts on human health.

## 5. Conclusions

As far as we know, this is the first report to link air pollution and human aspergillosis. Air pollution and influenza seasons correlate with all IPA, but not necessarily with influenza-associated IPA. The latter correlates with all subtypes of severe influenza. The significant correlation of IPA with PM or influenza was not universal in each year, probably influenced by an epidemic scale in influenza season, influenza A (H1N1), PM_2.5_, and PM_10_, which might have predominantly circulated in some years. We demonstrated the dynamic variation of effects of the influenza epidemic, influenza A (H1N1), and local ambient PMs during the northeast monsoon seasons on IPA annually. We postulated that some ambient fungal spores could contribute to the development of IPA, especially in vulnerable hosts during an influenza A (H1N1) epidemic. These can potentially aid in increasing the motivation of physicians to initiate the diagnosis of IPA, particularly in early stages of pneumonia during appropriate seasons. Public health measures, such as observing PM_2.5_ and PM_10_ in northeast monsoon seasons and early diagnosis may be helpful for potentially affected patients and for containing the fungal epidemiology.

## Figures and Tables

**Table 1:** Monthly cases of pulmonary aspergillosis and secular trend analysis from 2015 to 2019.

Year	2015 ^a^	2016 ^a^	2017 ^a^	2018 ^a^	2019 ^b^	*p* for mean
Mean	4.75	8.58	5.75	9.25	17.00	<0.0001
SD	1.82	9.20	3.19	3.74	5.05	
Slope	0	−0.394	0.134	−0.444	0.171	
CI 90%	(0, 0)	(−1.024, 0.236)	(−0.325, 0.593)	(−1.333, 0.444)	(−0.240, 0.583)	
*P* for slope	0.681	0.303	0.631	0.411	0.493	
Year	2015–2016		2015–2017	2015–2018	2015–2019	
Slope	0		0	0.077	0.185	
CI 90%	(0, 0)		(0, 0)	(0.021, 0.133)	(0.122, 0.248)	
*P* for slope	0.823		0.806	0.023	<0.00001	
Year	2015–2017			2018–2019		
Slope	0			0.500		
CI 90%	(0, 0)			(0.209, 0.791)		
*P* for slope	0.806			0.005		
Year	2015–2017 vs. 2018–2019					
Slope difference	−0.500					
CI 90%	(0, 0)					
*p* for slope difference	0.005					

a, retrospective data; b, prospective data; SD, standard deviation; CI, confidence interval.

**Table 2:** Spearman’s correlation between IPA case number and each variable, including all influenza, subtypes of influenza, and ambient pollution in Tainan.

IPA in 3 Tainan Institutes (*n*)	Influenza(all) (*n*, *p* Values)	Flu A (H3N2) (*n*, *p* Values)	Non-typed FluA (*n, p* Values)	Influenza B (*n, p* Values)	Flu A (H1N1) (*n*, *p* Values)	PM_2.5_ in Tainan	PM_10_ in Tainan
Retrospective (Stage 1)							
2015 (57)	92, 0.710	32, 0.479	32, 0.906	15, 0.526	13, 0.882	0.122	0.455
2016 (103)	235, 0.039	12, 0.654	94, 0.077	32, 0.089	97, 0.111	0.094	0.078
2017 (69)	133, 0.203	72, 0.627	56, 0.041 *	4, 0.466	1, 0.785	0.284	0.121
2018 (111)	89, 0.792	29, 0.257	20, 0.271	23, 0.220	17, 0.698	0.002	0.005
Subtotal (340)	549, 0.340	145, 0.335	202, 0.567	74, 0.301	128, 0.042	0.001	0.001
Prospective (Stage 2)							
2019 (204)	165, 0.198	27, 0.956	62, 0.724	2, 0.892	78, 0.015	0.334	0.552
Overall (544)	714, 0.002	171, 0.506	261, 0.124	76, 0.697	206, < 0.001	0.040	0.061

* negative correlation (*r_s_*, −0.596).

**Table 3:** Spearman’s correlation between % of IPA in severe influenza and each variable, including % of IPA in subtypes of severe influenza and ambient pollution in Tainan.

IPA in Severe Influenza (%, *n/*N)	IPA in Severe H1N1 (%, *n/*N)	IPA in Severe H3N2 (*%*, *n*/N)	IPA in Severe other Flu A (*%, n/*N)	IPA in Severe Flu B (%, *n/*N)	Tainan PM_2.5_	Tainan PM_10_
Retrospective (Stage 1)						
2015 (11.3, 7/62)	8.3, 1/12	10.7, 3/28	13.3, 2/15	14.3, 1/7	0.654	0.787
2016 (16,2, 22/136)	16.7, 13/78	0, 0/12	34.6, 9/26	0, 0/20	0.058	0.064
2017 (3.2, 2/62)	0, 0/0	1.8, 1/57	0, 0/2	33.3, 1/3	0.894	0.809
2018 (18.2, 10/55)	7.7, 1/13	11.1, 3/27	25, 1/4	45.5, 5/11	0.403	0.738
Subtotal (13.0, 41/315) *P* for 2015–2018	14.6, 15/103 0.007	5.6, 7/124 <0.001	25.5, 12/47 0.004	17.1, 7/41 <0.001	0.189	0.198
Prospective (Stage 2)						
2019 (21.7, 20/92) *P* for 2019	27.9, 19/68 <0.001	0/20 NA	33.3, 1/3 0.786	0/1 NA	0.221	0.427
Overall						
2015–2019 (15.0, 61/407)	19.9, 34/171	4.9, 7/144	26.0, 13/50	16.7, 7/42		
*P* for 2015–2019	<0.001	0.003	0.010	<0.001	0.201	0.203

Note. Other Flu A indicates non-H1N1, non-H3N2 severe influenza A, responsible for 18.4% (48/261) of non-typed Flu A (mostly influenza A without further identification).

**Table 4:** Predictors for IPA (2015–2019) by multiple linear regression.

	Univariate		Model A (Stepwise)		Model B (*p* < 0.1)		Model C (*p* < 0.05)	
Variables	β	*p*	β	*p*	β	*p*	β	*p*
Influenza (all)	0.21	<0.0001			0.34	0.0564	0.27	0.1158
H1N1	0.60	<0.0001	0.90	<0.0001	0.72	<0.0001	0.76	<0.0001
H3N2	–0.15	0.3717	0.25	0.1258				
Non-typed FluA	0.46	<0.0001	–0.44	0.0302	–0.92	0.0136	–0.82	0.0267
FluB	0.93	0.0034			–0.46	0.1302	–0.32	0.2761
Tainan PM_2.5_	0.14	0.0932	0.27	0.0845	0.09	0.1194		
Tainan PM_10_	0.06	0.2142	0.27	0.0161				
Zuoying PM_2.5_	0.06	0.4098	−0.28	0.1151				
Zuoying PM_10_	0.00	0.9259	−0.16	0.1397				
R square				0.6860		0.6072		0.5890

β, beta coefficients.

## Supplementary Materials

The following are available online at https://www.mdpi.com/2309-608X/7/3/227/s1, Figure S1: The secular trend (2015–2019) of PM_2.5_ (monthly average) in Tainan City (left) decreased but was statistically insignificant (*p* = 0.3387). In contrast, the trend of monthly cases of influenza A (H1N1) in the Chi Mei medical systems (right) significantly increased (slope, 0.0357; *p* = 0.001); Figure S2: A: Gradient of annually local ambient mean PM_2.5_ levels in 2018, increasing from northern to southern Taiwan. Epidemiology of aspergillosis in Tainan City was correlated with local ambient mean PM_2.5_ levels higher than 20 μg/m3 in areas south to central Taiwan. B: Tainan IPA in 2018 is significantly correlated with the local PM_2.5_ pollution (Tainan and Zuoying) in southern Taiwan, but not correlated with PM_2.5_ pollution in distant areas, like northern and eastern Taiwan, with an interface at central Taiwan between Xitun and Fengyuan districts; Figure S3: In 2018, Tainan IPA (cases/month) in Tainan City is significantly correlated with PM_10_ (μg/m3) at cities in southern Taiwan using Spearman’s correlation calculation, including Tainan west-central area (*p* = 0.005), Xinying (*p* = 0.0008), and Zuoying (*p* < 0.0001), but not related to distant areas in central Taiwan, such as Xituin (*p* = 0.064) and Fenyuan (*p* = 0.102) and in northern Taiwan, such as Taipei Chongshan district (*p* = 0.190). The vertical axis represents monthly case number for Tainan IPA and also for monthly average concentrations of PM_10_ of various areas; Figure S4: From 2015 to 2019, Tainan IPA monthly cases (blue bar) are positive correlated to local PM_2.5_ (μg/m3) curve in Tainan (red), but there is no statistically significant correlation of Tainan IPA with PM_2.5_ in distant areas, including Zuoying district of Kaohsiung City (black), Xitun district of Taichung City (yellow) and Chungshan District of Taipei City (green). The *p* values represent Spearman’s correlation calculation between Tainan IPA and PM_2.5_ of each area in various city.

## References

[1] Wu P.C., Tsai J.C., Li F.C., Lung S.C., Su H.J. (2004). Increased levels of ambient fungal spores in Taiwan are associated with dust events from China. Atmos. Environ..

[2] Cao C., Jiang W., Wang B., Fang J., Lang J., Tian G., Jiang J., Zhu T.F. (2014). Inhalable microorganisms in Beijing’s PM2.5 and PM10 pollutants during a severe smog event. Environ. Sci. Technol..

[3] Chao H.J., Chan C.-C., Rao C.Y., Lee C.-T., Chuang Y.-C., Chiu Y.-H., Hsu H.-H., Wu Y.-H. (2012). The effects of transported Asian dust on the composition and concentration of ambient fungi in Taiwan. Int. J. Biometeorol..

[4] Kallawicha K., Tsai Y.J., Chuang Y.C., Lung S.C.C., Wu C.D., Chen T.H., Chen P.C., Chompuchan C., Chao H.J. (2015). The spatiotemporal distributions and determinants of ambient fungal spores in the Greater Taipei area. Environ. Pollut..

[5] Guervilly C., Roch A., Ranque S., Forel J.-M., Hraiech S., Xeridat F., Adda M., Papazian L. (2012). A strategy based on galactomannan antigen detection and PCR for invasive pulmonary aspergillosis following influenza A (H1N1) pneumonia. J. Infect.

[6] Wauters J., Baar I., Meersseman P., Meersseman W., Dams K., De Paep R., Lagrou K., Wilmer A., Jorens P., Hermans G. (2012). Invasive pulmonary aspergillosis is a frequent complication of critically ill H1N1 patients: A retrospective study. Intensive Care Med..

[7] Schauwvlieghe A.F.A.D., Rijnders B.J.A., Philips N., Verwijs R., Vanderbeke L., Van Tienen C., Lagrou K., Verweij P.E., Van De Veerdonk F.L., Gommers D. (2018). Invasive aspergillosis in patients admitted to the intensive care unit with severe influenza: A retrospective cohort study. Lancet Respir. Med..

[8] Ku Y.H., Chan K.S., Yang C.C., Tan C.K., Chuang Y.C., Yu W.L. (2017). Higher mortality of severe influenza patients with probable aspergillosis than those with and without other coinfections. J. Formos. Med. Assoc..

[9] Yu W.-L., Liu W.-L., Chan K.-S., Yang C.-C., Tan C.-K., Tsai C.-L., Chen C.-M., Chuang Y.-C. (2018). High-level ambient particulate matter before influenza attack with increased incidence of *Aspergillus* antigenemia in southern Taiwan, 2016. J. Microbiol. Immunol. Infect..

[10] Schroeder M., Simon M., Katchanov J., Wijaya C., Rohde H., Christner M., Laqmani A., Wichmann D., Fuhrmann V., Kluge S. (2016). Does galactomannan testing increase diagnostic accuracy for IPA in the ICU? A prospective observational study. Crit. Care.

[11] Liu J.-W., Chen Y.-H., Lee W.-S., Lin J.-C., Chuang Y.-C., Lin H.-H., Liu Y.-C., Tang H.-J., Chen Y.-S., Ko W.-C. (2019). Randomized noninferiority trial of cefoperazone-sulbactam versus cefepime in the treatment of hospital-acquired and healthcare-associated pneumonia. Antimicrob. Agents Chemother..

[12] Donnelly J.P., Chen S.C., Kauffman C.A., Steinbach W.J., Baddley J.W., E Verweij P.E., Clancy C.J., Wingard J.R., Lockhart S.R., Groll A.H. (2020). Revision and update of the consensus definitions of invasive fungal disease from the European Organization for Research and Treatment of Cancer and the Mycoses Study Group Education and Research Consortium. Clin. Infect. Dis..

[13] Liu W.L., Yu W.L., Chan K.S., Yang C.C., Wauters J., Verweij P.E. (2019). Aspergillosis related to severe influenza: A worldwide phenomenon?. Clin. Respir. J..

[14] Chang S.Y., Fang G.C., Chou C.C., Chen W.N. (2006). Source identifications of PM_10_ aerosols depending on hourly measurements of soluble components characterization among different events in Taipei Basin during spring season of 2004. Chemosphere.

[15] Chuang M.T., Lee C.T., Hsu H.C. (2018). Quantifying PM_2.5_ from long-range transport and local pollution in Taiwan during winter monsoon: An efficient estimation method. J. Environ. Manag..

[16] Madhwal S., Prabhu V., Sundriyal S., Shridhar V. (2020). Ambient bioaerosol distribution and associated health risks at a high traffic density junction at Dehradun city, India. Environ. Monit. Assess..

[17] Liu Y., Wu J., Yu D., Ma Q. (2018). The relationship between urban form and air pollution depends on seasonality and city size. Environ. Sci. Pollut. Res. Int..

[18] Schwartz I.S., Friedman D.Z.P., Zapernick L., Dingle T.C., Lee N., Sligl W., Zelyas N., Smith S.W. (2020). High rates of influenza-associated invasive pulmonary aspergillosis may not be universal: A retrospective cohort study from Alberta, Canada. Clin. Infect. Dis..

[19] Thevissen K., Jacobs C., Holtappels M., Toda M., Verweij P., Wauters J. (2020). International survey on influenza-associated pulmonary aspergillosis (IAPA) in intensive care units: Responses suggest low awareness and potential underdiagnosis outside Europe. Crit. Care.

[20] Toda M., Beekmann S.E., Polgreen P.M., Chiller T.M., Jackson B.R., Beer K.D. (2020). Knowledge of infectious disease specialists regarding aspergillosis complicating influenza, United States. Emerg. Infect. Dis..

[21] Lai C.C., Chen C.M., Liao K.M., Chao C.M., Chan K.S., Yu W.L. (2021). A mysterious surge of aspergillosis among non-SARS-CoV-2 patients during COVID-19 pandemic. J. Microbiol. Immunol. Infect..

[22] Verweij P.E., Rijnders B.J.A., Brüggemann R.J.M., Azoulay E., Bassetti M., Blot S., Calandra T., Clancy C.J., Cornely O.A., Chiller T. (2020). Review of influenza-associated pulmonary aspergillosis in ICU patients and proposal for a case definition: An expert opinion. Intensive Care Med..

[23] Rozaliyani A., Sedono R., Jusuf A., Rumende C.M., Aniwidyaningsih W., Burhan E., Prasenohadi P., Handayani D., Yunihastuti E., Siagian F.E. (2019). A novel diagnosis scoring model to predict invasive pulmonary aspergillosis in the intensive care unit. Saudi Med. J..

