# Supplementary material for: Epidemiological Correlation of Pulmonary Aspergillus Infections with Ambient Pollutions and Influenza A (H1N1) in Southern Taiwan
Source: J Fungi (Basel). 2021 Mar 19;7(3):227. doi: 10.3390/jof7030227 (PMC8003483; doi:10.3390/jof7030227)
Supplement: Supplementary file 1 [file jof-07-00227-s001.pdf]

Supplement figures

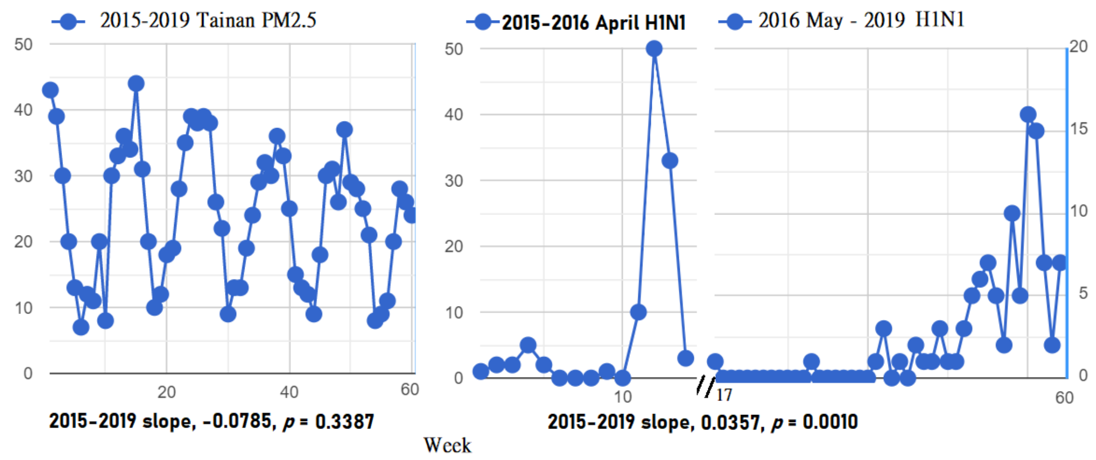

Supplementary Figure 1: The secular trend (2015-2019) of $PM_{2.5}$ (monthly average) in Tainan City (left) decreased but was statistically insignificant (p = 0.3387). In contrast, the trend of monthly cases of influenza A (H1N1) in the Chi Mei medical systems (right) significantly increased (slope, 0.0357; p = 0.001).

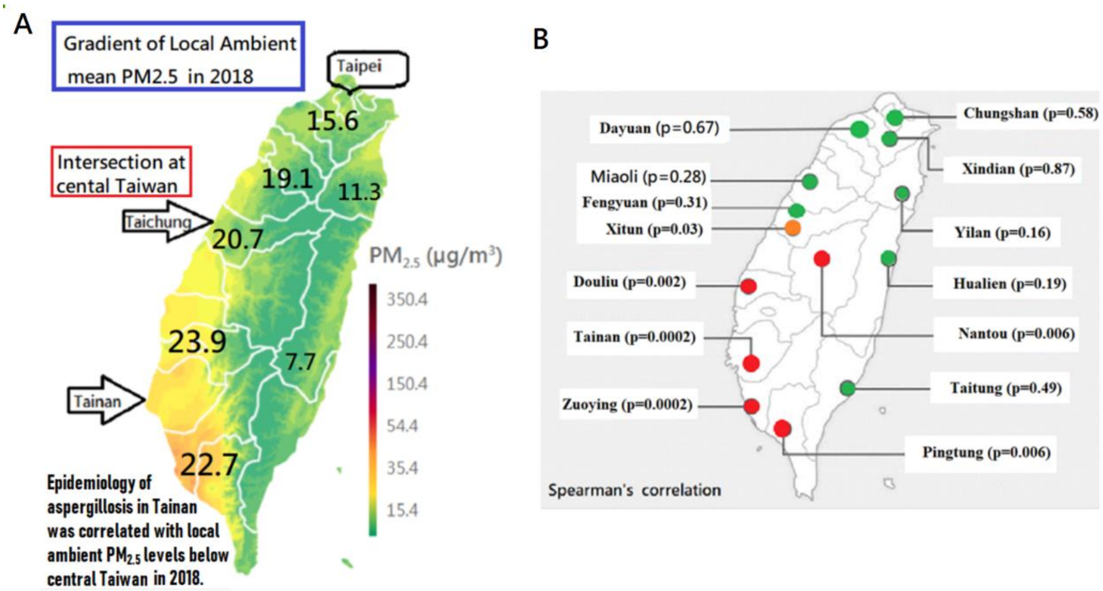

Supplementary Figure 2: A: Gradient of annually local ambient mean $PM_{2.5}$ levels in 2018, increasing from northern to southern Taiwan. Epidemiology of aspergillosis in Tainan City was correlated with local ambient mean $PM_{2.5}$ levels higher than 20 μg/m^3 in areas south to central Taiwan. **B:** Tainan IPA in 2018 is significantly correlated with the local PM2.5 pollution (Tainan and Zuoying) in southern Taiwan, but not correlated with PM2.5 pollution in distant areas, like northern and eastern Taiwan, with an interface at central Taiwan between Xitun and Fengyuan districts.

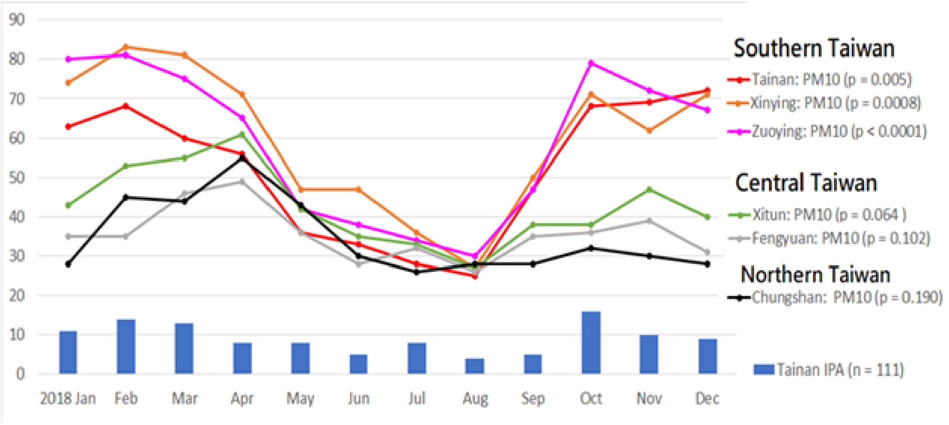

Supplementary Figure 3: In 2018, Tainan IPA (cases/month) in Tainan City is significantly correlated with PM_{10} ($\mu g/m^3$) at cities in southern Taiwan using Spearman's correlation calculation, including Tainan west-central area (p = 0.005), Xinying (p = 0.0008), and Zuoying (p < 0.0001), but not related to distant areas in central Taiwan, such as Xituin (p = 0.064) and Fenyuan (p = 0.102) and in northern Taiwan, such as Taipei Chongshan district (p = 0.190). The vertical axis represents monthly case number for Tainan IPA and also for monthly average concentrations of PM_{10} of various areas.

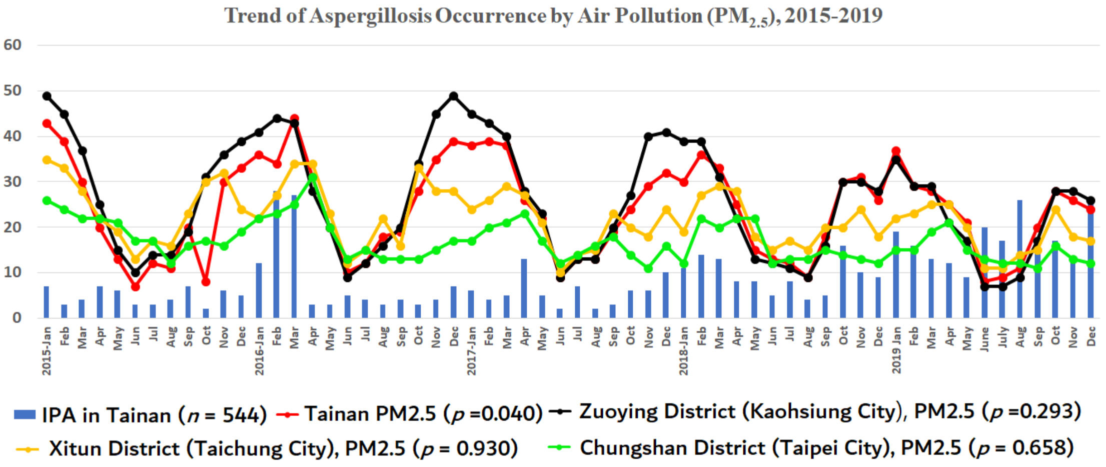

Supplementary Figure 4: From 2015 to 2019, Tainan IPA monthly cases (blue bar) are positive correlated to local $PM_{2.5}$ ($\mu g/m^3$) curve in Tainan (red), but there is no statistically significant correlation of Tainan IPA with $PM_{2.5}$ in distant areas, including Zuoying district of Kaohsiung City (black), Xitun district of Taichung City (yellow) and Chungshan District of Taipei City (green). The p values represent Spearman's correlation calculation between Tainan IPA and $PM_{2.5}$ of each area in various city.
